# Knowledge gaps in dialysis: from recognition to implementation of intradialytic exercise

**DOI:** 10.1590/2175-8239-JBN-2026-E002en

**Published:** 2026-01-16

**Authors:** Fernanda Salomão Gorayeb-Polacchini

**Affiliations:** 1 Sociedade Brasileira de Nefrologia, São Paulo, SP, Brazil.; 2 Hospital de Base de São José do Rio Preto, São José do Rio Preto, SP, Brazil.

As the number of patients on dialysis grows, debilitating conditions such as sarcopenia and muscle atrophy have become constant concerns in nephrology practice. In patients on renal replacement therapy (RRT)—both on dialysis and after kidney transplantation—exercising has moved beyond a mere adjuvant strategy to become part of person-centered care, an essential component of contemporary nephrology. Structured exercise programs in this population are associated with improvements in physical and cardiovascular capacity, nutritional parameters, and quality of life^1^.

There is evidence that structured intradialytic exercise programs, at light to moderate intensity—aerobic, resistance, or combined (e.g., cycling, bands/elastic bands, and weights)—improve cardiovascular performance, blood pressure control, and tolerance to ultrafiltration. They also increase functional capacity and muscle strength; attenuate depressive symptoms; reduce serum phosphorus levels; and have a favorable safety profile. In centers that incorporate the practice of exercise into routine care, additional organizational gains are observed, such as greater staff engagement and a better patient experience^2–6^.

The *I Brazilian Guideline on Hypertension in Dialysis*, from the Brazilian Society of Nephrology (SBN), recommends the practice of intradialytic physical activity or during the interdialytic interval and, whenever possible, under the supervision of physical educators and/or physical therapists in dialysis units^7^.

In Brazil, however, the mismatch between evidence, regulation, and financing persists. The current regulation (ANVISA RDC No. 11/2014) defines the minimum team for dialysis services, which does not include physical therapists or physical educators^8^. In practice, this gap discourages the implementation of exercise programs during dialysis, even in the face of evident benefit^2,3,4,5,6,7^. In parallel, a national study commissioned by the SBN showed a 38% shortfall in hemodialysis reimbursement relative to the real cost per session, with a direct impact on the sustainability of services (closure of clinics and hospitalizations due to lack of slots)^9^. The funding shortfall puts pressure on supplies, human resources, energy, and equipment maintenance, reducing the margin for innovation precisely where it would most affect outcomes and patient experience. The combined effect is predictable: even where there is clinical leadership and scientific evidence, there is a lack of legal backing and budgetary space to routinely incorporate intradialytic physical exercise.

In this context, the study “Brazilian nephrologist’s knowledge of intradialytic exercise: a national survey” by Cordeiro et al.^10^ offers a timely contribution. From a nationally representative sample of nephrologists, the study maps training gaps and perceived barriers to the practice of intradialytic exercise, incorporating methodological and scope innovation. The survey revealed a structural educational deficit (65.5% without formal training on the topic) and a direct relationship between beliefs, knowledge, and conduct: professionals who endorse intradialytic exercise are more likely to recognize clinical impact and recommend it. Interestingly, more years in practice were associated with less formal exposure to the subject—a clear sign of the need for targeted continuing education.

By quantifying gaps previously described anecdotally and demonstrating that beliefs and knowledge modulate clinical recommendation, the authors shift the debate from “whether it works” to “how to make it work here”^10^. Despite the limitations inherent to cross-sectional studies, including self-report and selection bias, the evidence generated is nonetheless informative and operationally useful^10^.

The findings are relevant to improving the care process in several aspects. (a) Education: residency curricula, continuing education courses, and internal trainings should include intradialytic exercise (screening, contraindications, load progression, and event monitoring), with a particular focus on more experienced nephrologists, in whom the informational gap was more evident^10^. (b) Protocols: the need for standardized, feasible, low-cost protocols. (c) Clinical governance: definition and monitoring of minimum metrics—eligibility, per-session adherence, adverse events, variation in functional capacity, and patient satisfaction. (d) Regulation and financing: national data provide a concrete basis for dialogue with policymakers.

Recognizing the importance of physical therapists and/or physical educators in the minimum team composition and adjusting funding to include intradialytic rehabilitation programs should not only be optional increments, but also decisions of technical and allocative efficiency, consistent with value-based care. Moreover, performing exercise during the dialysis session optimizes resources and the patient’s time, promoting greater adherence and a better care experience. Until guidelines are formalized, the adoption of feasible protocols and continuing medical education for nephrologists is proposed.

In summary, integrating intradialytic exercise emerges as a rare opportunity to deliver better outcomes and experience, with modest incremental investment, when anchored in well-structured protocols, a trained team, and routine outcome evaluation. The article by Cordeiro et al.^10^ provides a national, actionable lever to advance the dimensions of educating, standardizing, measuring, and advocating, turning the dialysis room into a space for effective rehabilitation as well.

## Data Availability

No new data were generated or analyzed in this study.
