# Supplementary material for: Knowledge gaps in dialysis: from recognition to implementation of intradialytic exercise
Source: J Bras Nefrol. 2026 Jan 16;48(1):e2026E002. doi: 10.1590/2175-8239-JBN-2026-E002en (PMC12817534; doi:10.1590/2175-8239-JBN-2026-E002en)
Supplement: Translation

# Translation: Lacunas de conhecimento em diálise: do reconhecimento à implementação do exercício intradialítico

**DOI:** 10.1590/2175-8239-JBN-2026-E002pt

**Authors:** Fernanda Salomão Gorayeb-Polacchini

**Affiliations:** 1 Sociedade Brasileira de Nefrologia, São Paulo, SP, Brasil.; 2 Hospital de Base de São José do Rio Preto, São José do Rio Preto, SP, Brasil.

À medida que o número de pacientes em diálise cresce, condições debilitantes como sarcopenia e atrofia muscular tornaram-se preocupações constantes na prática do nefrologista. Em pacientes em Terapia Renal Substitutiva (TRS) — tanto em diálise quanto após o transplante renal — o exercício físico deixou de ser mera estratégia adjuvante para integrar o cuidado centrado na pessoa, componente essencial da nefrologia contemporânea. Programas estruturados de exercício nessa população associam-se à melhora da capacidade física e cardiovascular, de parâmetros nutricionais e da qualidade de vida^1^.

Há evidências de que programas estruturados de exercício intradialítico, em intensidade leve a moderada — aeróbicos, resistidos ou combinados (p. ex., ciclismo, faixas/elásticos e pesos) — melhoram o desempenho cardiovascular, o controle pressórico e a tolerância à ultrafiltração. Também aumentam a capacidade funcional e a força muscular; atenuam sintomas depressivos; reduzem níveis séricos de fósforo; e apresentam bom perfil de segurança. Em centros que incorporam essa prática à rotina, observam-se ainda ganhos organizacionais, como maior engajamento da equipe e melhor experiência do paciente^2–6^.

A *I Diretriz Brasileira de Hipertensão Arterial na Diálise*, da Sociedade Brasileira de Nefrologia (SBN), recomenda a prática de atividade física intradialítica ou no intervalo interdialítico e, sempre que possível, sob supervisão de profissionais de educação física e/ou fisioterapeutas nas unidades de diálise^7^.

No Brasil, contudo, persiste o descompasso entre evidências, regulação e financiamento. A regulamentação vigente (RDC ANVISA nº 11/2014) define a equipe mínima para os serviços de diálise, não incluindo fisioterapeutas ou educadores físicos^8^. Na prática, essa lacuna desestimula a implementação de programas de exercício durante a diálise, mesmo diante de evidências de benefício^2–7^.

Em paralelo, um estudo nacional encomendado pela SBN evidenciou defasagem de 38% no repasse financeiro da hemodiálise em relação ao custo real da sessão, com impacto direto na sustentabilidade dos serviços (fechamento de clínicas e internações por ausência de vagas)^9^. A defasagem de custeio na diálise pressiona insumos, recursos humanos, energia e manutenção de equipamentos, reduzindo a margem para inovação assistencial justamente onde ela mais impactaria os desfechos e experiência do paciente. O efeito combinado é previsível: mesmo onde há liderança clínica e evidência científica, faltam respaldo legal e espaço orçamentário para incorporar rotineiramente o exercício físico intradialítico.

É nesse cenário que o estudo “Conhecimento de nefrologistas brasileiros sobre exercícios intradialíticos: uma pesquisa nacional”, de Cordeiro et al.^10^, oferece contribuição oportuna. A partir de uma amostra representativa de nefrologistas de todo o país, o trabalho mapeia lacunas formativas e barreiras percebidas à prática do exercício intradialítico, incorporando inovação metodológica e de escopo. A pesquisa evidenciou déficit educacional estruturante (65,5% sem formação formal no tema) e relação direta entre crenças, conhecimento e conduta: profissionais que endossam o exercício intradialítico têm maior probabilidade de reconhecer impacto clínico e recomendá-lo. Curiosamente, mais anos de atuação associaram-se a menor exposição formativa ao assunto — sinal claro da necessidade de educação continuada dirigida.

Ao quantificar lacunas antes descritas de forma anedótica e demonstrar, que crenças e conhecimento modulam a recomendação clínica, os autores deslocam o debate de “se funciona” para “como fazer funcionar aqui”^10^. Apesar das limitações inerentes a estudos transversais, incluindo o autorreporte e viés de seleção; ainda assim, a evidência gerada é informativa e operacionalmente útil^10^.

Os achados são relevantes para a melhoria do processo assistencial por várias razões. (a) Educação: currículos de residência, cursos de atualização e treinamentos internos devem incluir o exercício intradialítico (triagem, contraindicações, progressão de carga e monitoramento de eventos), com foco especial em nefrologistas mais experientes, nos quais a lacuna informacional foi mais evidente^10^. (b) Protocolos: necessidade de protocolos padronizados, factíveis e de baixo custo. (c) Governança clínica: definição e monitoramento de métricas mínimas — elegibilidade, adesão por sessão, eventos adversos, variação de capacidade funcional e satisfação do paciente. (d) Regulação e financiamento: dados nacionais conferem base concreta para o diálogo com formuladores de políticas.

Reconhecer fisioterapeutas e/ou profissionais de educação física na composição mínima da equipe e ajustar o custeio incluindo os programas de reabilitação intradialítica, não deve ser incrementos facultativos, mas decisões de eficiência técnica e alocativa, coerentes com o cuidado baseado em valor. Ademais, a realização do exercício durante a sessão de diálise otimiza recursos e tempo do paciente, promovendo maior adesão e melhor experiência do cuidado. Até a formalização de diretrizes, propõe-se a adoção de protocolos viáveis e educação médica continuada para nefrologistas.

Em síntese, a integração do exercício intradialítico desponta como oportunidade rara de entregar melhores desfechos e experiência, com investimento incremental modesto, quando ancorada em protocolos bem estruturados, equipe capacitada e avaliação rotineira de resultados. O artigo de Cordeiro et al.^10^ fornece a alavanca nacional e acionável para avançar nas dimensões educar, padronizar, medir e defender, convertendo a sala de diálise também em espaço de reabilitação efetiva.

## Data Availability

Nenhum dado novo foi gerado ou analisado neste estudo.
